# The Clinical Impact of Sample Storage at −20 °C on Renin Reference Intervals and Aldosterone–Renin Ratio Calculations

**DOI:** 10.1210/clinem/dgae057

**Published:** 2024-01-30

**Authors:** Ömer Özcan, Jacquelien J Hillebrand, Wendy den Elzen, Annemieke C Heijboer

**Affiliations:** Endocrine Laboratory, Department of Laboratory Medicine, Amsterdam UMC location University of Amsterdam, 1105 AZ Amsterdam, The Netherlands; Amsterdam Gastroenterology, Endocrinology & Metabolism, 1105 AZ Amsterdam, The Netherlands; Endocrine Laboratory, Department of Laboratory Medicine, Amsterdam UMC location University of Amsterdam, 1105 AZ Amsterdam, The Netherlands; Amsterdam Gastroenterology, Endocrinology & Metabolism, 1105 AZ Amsterdam, The Netherlands; Endocrine Laboratory, Department of Laboratory Medicine, Amsterdam UMC location University of Amsterdam, 1105 AZ Amsterdam, The Netherlands; Laboratory Specialized Diagnostics & Research, Department of Laboratory Medicine, Amsterdam UMC, location University of Amsterdam, 1105 AZ Amsterdam, The Netherlands; Amsterdam Public Health Research Institute, 1105 AZ Amsterdam, Netherlands; Endocrine Laboratory, Department of Laboratory Medicine, Amsterdam UMC location University of Amsterdam, 1105 AZ Amsterdam, The Netherlands; Amsterdam Gastroenterology, Endocrinology & Metabolism, 1105 AZ Amsterdam, The Netherlands; Department of Laboratory Medicine, Amsterdam UMC location Vrije Universiteit Amsterdam, 1081 HZ Amsterdam, The Netherlands; Amsterdam Reproduction & Development Research Institute, 1105 AZ Amsterdam, The Netherlands

**Keywords:** renin, preanalysis, cryoactivation, laboratory, hypertension, aldosterone

## Abstract

Cryoactivation is known to occur in whole blood and plasma samples when kept between +4 and −5 °C, leading to falsely high renin concentrations. In 2022 it has been clearly shown that cryoactivation can also occur in samples stored at −20 °C. Based on these new findings, here we discuss how this can influence the clinical diagnosis of patients. First, we show that storage of renin plasma samples can affect the renin measurements and thereby the aldosterone to renin ratio (ARR) calculation, which might explain the high intraindividual variability in ARR also recently demonstrated. Second, we discuss the existing studies on the establishment of renin reference intervals and note the lack of attention given to this recently revealed preanalytical condition. Our literature review of the reference intervals for renin suggest that cryoactivation might have influenced the published data.

Historically, the determination of renin levels involved activity assays, for which blood samples were collected in precooled tubes and then centrifuged in a cooled environment (1, 2). The extracted plasma was either stored in a cooled state or frozen until analysis, with thawing performed at 4 °C. However, the discovery of cryoactivation, which leads to conversion of prorenin to renin when samples are exposed to temperatures between −5 and +4 °C for extended periods of time (3-7), has raised doubts about these protocols. It is now recognized that preventing preanalytical cryoactivation of renin is crucial to ensure accurate results when using renin activity or mass assays.

In 2016, the Endocrine Society published the clinical practice guideline (8) on primary aldosteronism (PA), which serves as a comprehensive consensus paper on renin and aldosterone testing. Although primarily targeted at clinicians managing PA, it also provides valuable guidance for laboratories. The guidelines provide certain preanalytical recommendations for renin, such as transporting blood samples at ambient temperature instead of cooling them prior to centrifugation, and rapidly freezing and storing the plasma in a frozen state before testing. Remarkably, a recent study has revealed that storing samples in standard −20 °C freezers, as opposed to −70 °C or −80 °C freezers, can also lead to significantly higher (over 300%) direct renin measurement results (9). Furthermore, another study showed that plasma freezing time can vary depending on the type of −20 °C freezer used, potentially increasing measured renin concentrations (10). In our laboratory, we confirmed the presence of falsely high renin concentrations in EDTA plasma samples which were frozen and stored in −20 °C compared with −80 °C freezers. This means that in addition to storage between −5 °C and 4 °C, also freezing and storing samples at −20 °C can lead to cryoactivation and thereby to falsely high renin concentrations, which can have major effects on clinical interpretation.

In this opinion paper, we discuss the potential influence of cryoactivation due to storage at −20 °C on clinical interpretation of renin concentrations. First, by coincidence, we experienced unusual high direct renin concentrations when repeating direct renin measurements in our routine diagnostic setting. When measuring direct renin concentrations in plasma samples (immediately frozen after workup at −20 °C) and repeating measurements in their spare aliquots (also immediately frozen at −20 °C) after 1 week, we noted remarkable differences in renin concentrations of initial and spare aliquots of some samples. We measured, for instance, initial renin concentrations of 68 and 96 mU/L, whereas repeated measurements in the spare plasma aliquots were 762 and 13 mU/L respectively (with a reference interval [RI] of <45 mU/L; Liaison XL, Diasorin). Substantial differences in renin results (here 1021% and 87%, due to presumably cryoactivation in the initial or spare aliquot respectively) upon storage at −20 °C will also significantly impact aldosterone to renin ratio (ARR) calculations. Ng et al (11) recently emphasized that huge variations of the ARR in screening for PA are still a problem in clinical diagnostics. Unfortunately, the potential impact of renin cryoactivation was not mentioned in their article, showing that sample collection and storage conditions may represent a significant but overlooked factor contributing to the variability in renin concentration measurements and also ARR calculations. Future studies investigating ARR variability should consider the influence of cryoactivation on renin measurements and thereby ARR.

To ensure reliable and clinically relevant renin levels and ARR for screening (and diagnosis) of PA, it is recommended that studies on renin and ARR measurements provide detailed information regarding sample collection and storage conditions (temperature and duration). By taking appropriate precautions to prevent cryoactivation through correct sample handling and storage, it is expected that intraindividual and interindividual variations in renin measurements will be reduced, leading to enhanced reliability and clinical utility of the ARR in PA diagnosis.

Moreover, even when samples for renin measurements are stored at −80 °C, the RI used by the laboratories may be determined based upon experiments with inappropriate preanalytical storage conditions. To investigate how RI studies have been executed since 2016, we conducted a search in PubMed for studies defining renin RIs, focusing on those studies’ sample collection, transport, and storage conditions. We identified 9 studies defining RIs, in different age groups, for renin concentration or activity. Most of the studies utilized room temperature during sample handling and centrifugation (12-15), while only 4 studies mentioned the temperature at which the samples were stored before analyses and none of the studies provided information regarding the conditions of sample transport (see Table 1). Three studies did not give any information about sample preparation, transport, and storage conditions (16-18). Only 2 of the 9 studies (14, 19) stored plasma samples in −80 °C freezers; in 1 study the samples were handled for 8 hours under unspecified conditions before freezing (19), while in the other study the samples were immediately frozen and stored at −80 °C (14). Consequently, only 1 study was described to have avoided the potential cryoactivation effect. Interestingly, this particular study reported the lowest upper limit of the RI at 20.1 mIU/L (14), in contrast to the other studies using the same analytical method with upper limits ranging between 58.3 (17) and 99.8 mIU/L (12). These findings support the notion that using previous guidelines for preanalytical sample handling procedures in renin measurements may result in erroneously high upper limits of the RI with subsequently high impact on clinical interpretation.

**Table 1.** Studies defining renin concentration and renin activity reference intervals since 2016

	Study population	Direct Renin Concentration		Renin Activity		Preanalytical conditions		
		Reference interval	Method	Reference interval	Method	Sample preparation	Transport	Storage
Gibbons et al (2021) (20)	110 healthy volunteers (>18 years)	—	—	0.2-3.7 ng/mL/hour	LC-MS/MS	N.I.	N.I.	Freezer (temperature was not stated)
Yin et al (2023) (15)	309 healthy volunteers (>18 years)	—	—	0.10-3.23 ng/mL/hour	LC-MS/MS	Separated at RT	N.I.	N.I.
Lewandowski et al (2022) (16)	70 healthy pregnant women (age 30.53 ± 4.51 years) and 22 nonpregnant healthy women (age 33.08 ± 8.72 years)	6.25-59.36 µIU/mL and 11.12-82.55 µU/mL, respectively	Chemiluminescence immunoassay Liaison (Diasorin)	—	—	N.I.	N.I.	N.I.
Then et al (2022) (14)	2931 volunteers (56.2 ± 13.2 years age)	6.2-20.1 µU/mL	Chemiluminescence immunoassay Liaison (Diasorin)	—	—	Separated at RT	N.I.	−80 °C
Pastén et al (2022) (17)	54 normotensive pregnant women at term gestation	34.9-58.3 µU/mL	Chemiluminescence immunoassay Liaison (Diasorin)	—	—	N.I.	N.I.	N.I.
Deng et al (2018) (12)	328 healthy volunteers (18-73 ages)	Males: 3.3-92.7 µU/mL, Females: 3.7-99.8 µU/mL	Chemiluminescence immunoassay Liaison (Diasorin)	—	—	Separated at RT within 1 hours	RT	−20 °C
Miller et al (2023) (19)	48 healthy and metabolically stable babies (<3 years) and 119 children and adolescents (3 to <19 years)	7.7-171.5 µIU/mL (<3 years) and 9.4-93.0 µU/mL (3-18 years)	Chemiluminescence immunoassay Liaison (Diasorin)	—	—	Separated and aliquoted within 8 hours of collection (no temperature data)	N.I.	−80 °C
O'Shea et al (2016) (13)	266 healthy volunteers (>18 years)	6.8-86.6 µU/mL	Chemiluminescence immunoassay iSYS (IDS)	—	—	Separated at RT, within 30 minutes	RT	−20 °C
Xu et al (2023) (18)	262 preterm neonates	1 054-2818 mIU/L	Chemiluminescence immunoassay Liaison (Diasorin)	—	—	N.I.	N.I.	N.I.

Abbreviations: LC-MS/MS, liquid chromatography tandem mass spectrometry; N.I., no information; RT, room temperature.

In conclusion, it is crucial to consider the positive interference of cryoactivation on renin concentration and activity measurements, even under −20 °C storage conditions, both when measuring patient blood samples and when establishing accurate RIs, as both can influence the clinical interpretation. To prevent preanalytical interference caused by cryoactivation on renin measurements, we recommend performing blood collection, centrifugation, and handling at room temperature. Additionally, as renin in plasma at room temperature is stable for a maximum of 3 days (9), plasma samples that will be stored for longer than 3 days should be stored under conditions of −70 °C or −80 °C before renin analyses will take place. In these cases, samples should be transported on dry ice to make sure that samples remain frozen. Hence it is of utmost importance for clinical diagnostics that all study protocols and (research) papers are supplied with detailed information about the sample handling, transport, and storage temperature conditions considering renin concentration or activity measurements.

## Data Availability

Data sharing is not applicable to this article as no datasets were generated or analyzed during the current study.

## Abbreviations

ARR: aldosterone to renin ratio
PA: primary aldosteronism
RI: reference interval
